# Supplementary material for: Ultrafast charge-transfer-induced spin transition in cobalt-tungstate molecular photomagnets
Source: Nat Commun. 2025 Jun 6;16:5012. doi: 10.1038/s41467-025-60401-4 (PMC12144094; doi:10.1038/s41467-025-60401-4)
Supplement: Supplementary file 1 — Supplementary Information [file 41467_2025_60401_MOESM1_ESM.pdf]

SUPPLEMENTARY INFORMATION

Ultrafast charge-transfer-induced spin transition in cobalt-tungstate molecular photomagnets

Kazuki Nakamura,[a,b] Laurent Guérin,[b,c] Gaël Privault,[b,c] Koji Nakabayashi,[a,b*] Marius Hervé, [b,c] Eric Collet,[b,c,d*] and Shin-ichi Ohkoshi[a,b*]

[a] *Department of Chemistry, School of Science, The University of Tokyo 7-3-1 Hongo, Bunkyo-ku, Tokyo 113-0033, Japan.*

[b] *DYNACOM IRL2015 University of Tokyo - CNRS - Universite de Rennes, Department of Chemistry, 7-3-1 Hongo, Bunkyo-ku, Tokyo 113-0033, Japan.*

[c] *Univ Rennes, CNRS, IPR (Institit de Physique) - UMR 6251, 35000 Rennes, France.*

[d] *Institut Universitaire de France (IUF), Paris, France.*

*To whom correspondence should be addressed
E-mail: knakabayashi@chem.s.u-tokyo.ac.jp,
eric.collet@univ-rennes.fr, ohkoshi@chem.s.u-tokyo.ac.jp

Table of Contents

Supplementary Note 1: Characterization of CsCoW and CoW 2
Supplementary Note 2: Experimental configuration 3
Supplementary Note 3: UV-vis-NIR spectra of CsCoW in PIPT at low temperature 5
Supplementary Note 4: Photomagnetism of CsCoW 6
Supplementary Note 5: Sub-ps time delay ΔOD map of CsCoW at 230 K and 100 K 7
Supplementary Note 6: Data analysis of sub-ps time measurements 8
Supplementary Note 7: DFT calculation 14
Supplementary Note 8: Data analysis of long-picosecond time scale 18
Supplementary Note 9: Gaussian fitting for spectral trace 19
Supplementary Note 10: Fluence dependence at RT 22

Supplementary Note 1: Characterization of CsCoW and CoW

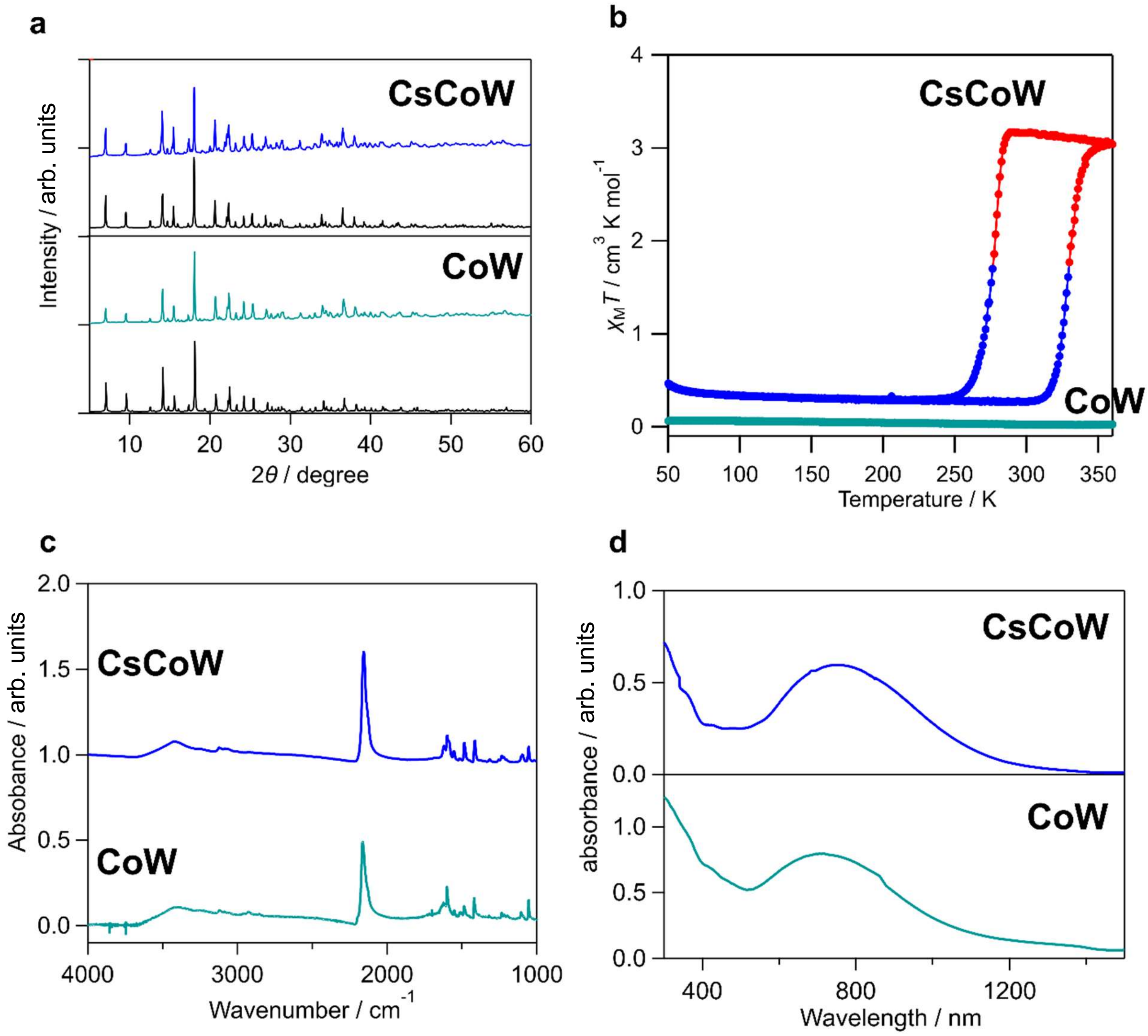

Supplementary Fig. 1. Characterization of CsCoW and CoW. a PXRD pattern for CsCoW (top) and CoW (bottom) at RT. The blue and light blue indicate experimental data for CsCoW and CoW. Black lines present calculated patterns. **b** $\chi_M T$-T plots under 1000 Oe. The Light blue is the CoW in the LT state and the other dataset corresponds to the CsCoW in the LT (blue) and HT (red) state. **c** IR spectra at RT. **d** UV-vis spectra at RT. The blue and light blue lines show the LT state of CsCoW and CoW, respectively.

Supplementary Note 2: Experimental configuration

The photoinduced dynamics were studied with a time-resolved experiment based on the typical pump-probe technique. First, an amplified Ti:sapphire femtosecond laser (Astrella) delivers a powerful 800 nm beam (7 mJ, 65 fs) at 1 kHz repetition rate, which is the core of the optical setup. Then, this pulse is split in two to seed the pump and probe beams. Regarding the pump pulse, we first tune the beam wavelength from 800 nm to 850 nm with an optical parametric amplifier. Then, the pump repetition rate is set to 500 Hz, by an optical chopper, to enable the shot-to-shot detection system. Regarding the probe, the supercontinuum is generated through a sapphire plate and covers the full visible range. This pulse is then homogeneously focalized by a silver parabolic mirror on the sample, with a beam size of 150x150 μm^2 (FWHM) measured by the typical knife-edge technique. Whereas the pump is focalized (450x450 μm^2) by a lens and pass through the parabolic mirror, resulting in only a few degrees of angle difference between both beams. After the sample, the pump beam is cut by an iris and a long pass filter 750 nm. Finally, for the detection system, the supercontinuum is collected by a coupler and send on a spectrometer (Princeton Instruments ACTION SP2500). The grating selectively spread out the probe spatially as a function of the wavelength, which is then read by a 2D CMOS detector (Basler aca2440-20gm). Moreover, as the probe repetition rate of the camera is set the same as the probe (1 kHz), we can record the photoinduced dynamics between each two consecutive shots, this for multiple images (from 1000 to 20000) and multiple trips (1 to 15). Overall, this experimental configuration enables to perform ultrafast time-resolved measurements with 65 femtosecond time resolution, on a broadband wavelength region spanning from 500 to 750 nm, with a shot-to-shot detection system and reaching, at best, a noise floor of 1×10^{-5}.

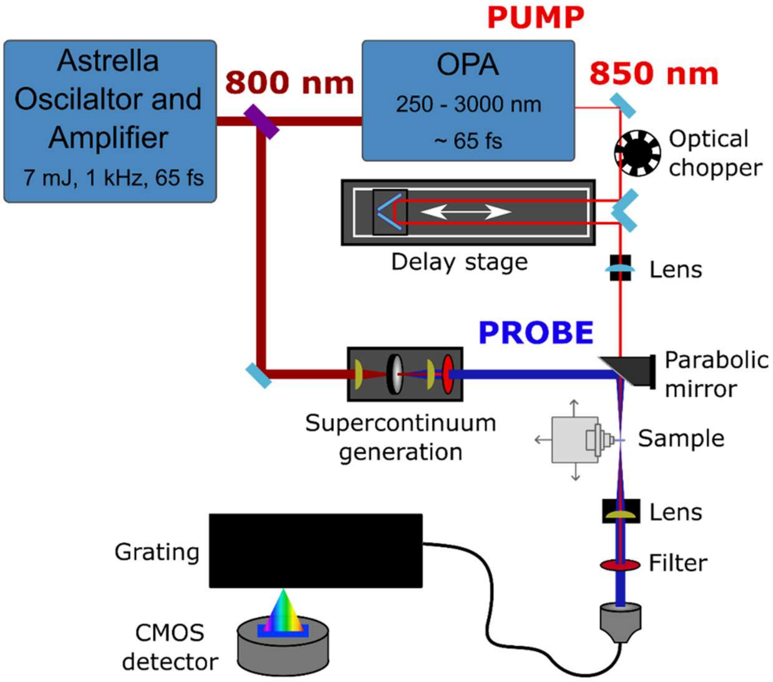

Supplementary Fig. 2. Schematic representation of the time-resolved optical layout.

For sub-ps timescales, the sample contribution signal data were obtained in two steps: First we corrected the pulse chirping of each 2D time-resolved maps, for the raw measurements with the samples (CsCoW or CoW) as presented in the example of Supplementary Fig. 3a1, and for the substrate (only the glass and liquid paraffin) as displayed in Supplementary Fig. 3b1. This correction is using a simple quadratic polynomial function to fit the maximum signal of the time-zero artefact from each wavelength. The corrected maps are then shown in Supplementary Fig. 3a2 and 3b2 for the sample and the substrate, respectively. The second step corresponds to the removal of the time-zero artefact, we subtracted Supplementary Fig. 3b2 to 3a2 and obtained 3c map, after scaling the substrate 2D map to the sample's map (from 0.8 to 1.1). One of the reason behind this scaling might be due to difference between the sample and substrate thickness.

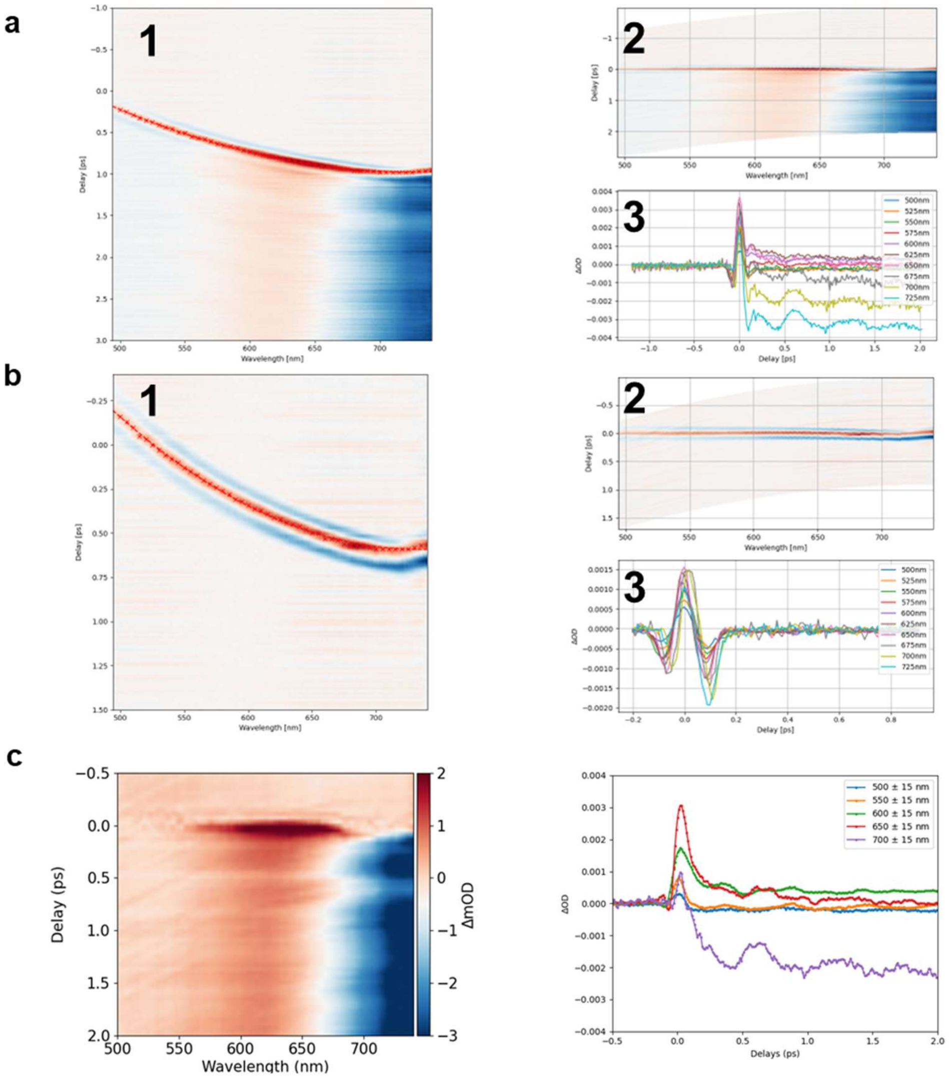

Supplementary Fig. 3. Raw data treatment. a, b Chirp correction for sample and substrate, respectively. The data of CsCoW at RT is employed as example. (1) Transient 2D map uncorrected. (2) Chirp corrected 2D maps. (3) Time trace for multiple wavelengths (in legends) after chirp correction. **c** Resultant 2D map after subtraction (left) and corresponding time trace (right).

Supplementary Note 3: UV-vis-NIR spectra of CsCoW in PIPT at low temperature

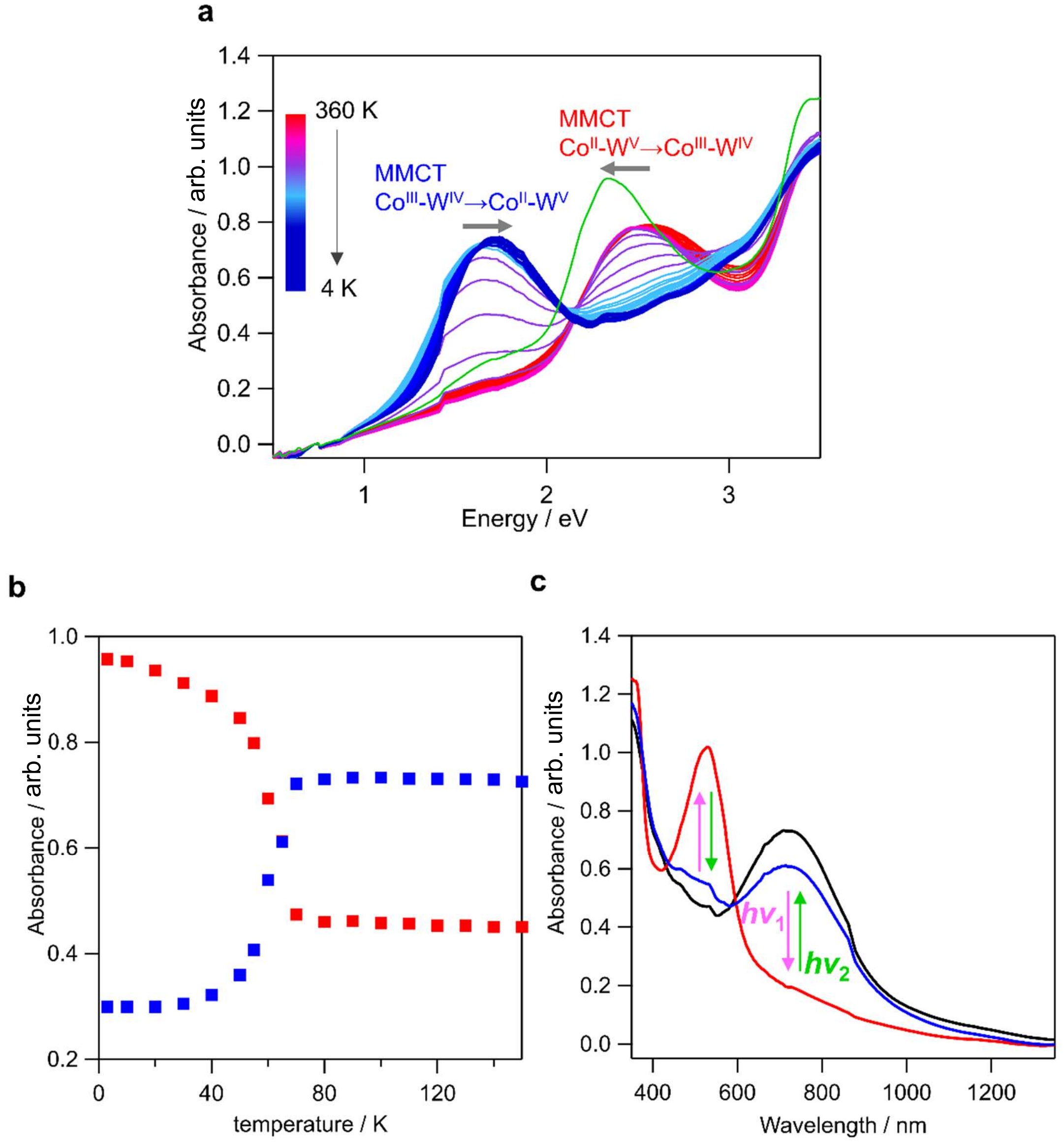

Supplementary Fig. 4. UV-vis-NIR spectra in PIPT at low temperature. a Comparison of the thermal and PIPT absorption spectra. Red to blue lines present the temperature dependence experimental data from 360 K to 4 K, and green line show the PI state at 4 K after photo irradiation (785 nm, 160 mW cm^{-2}). Each state shows the energy shift by temperature, and the PI state located the lower energy than HT state. **b** Thermal relaxation after photo irradiation in the UV-vis spectra. Red and blue squares indicate the absorption intensity of the peak at 530 nm and 760 nm, respectively. **c** Reverse-CTIST in the UV-vis spectra. Absorption spectra of CsCoW: in the ground LT at 4 K (black), after photo irradiation (red) by a cw laser at 785 nm (160 mW cm^{-2}) from LT to PI state, and after photo irradiation at 532 nm (145 mW cm^{-2}) from PI to LT state.

Supplementary Note 4: Photomagnetism of CsCoW

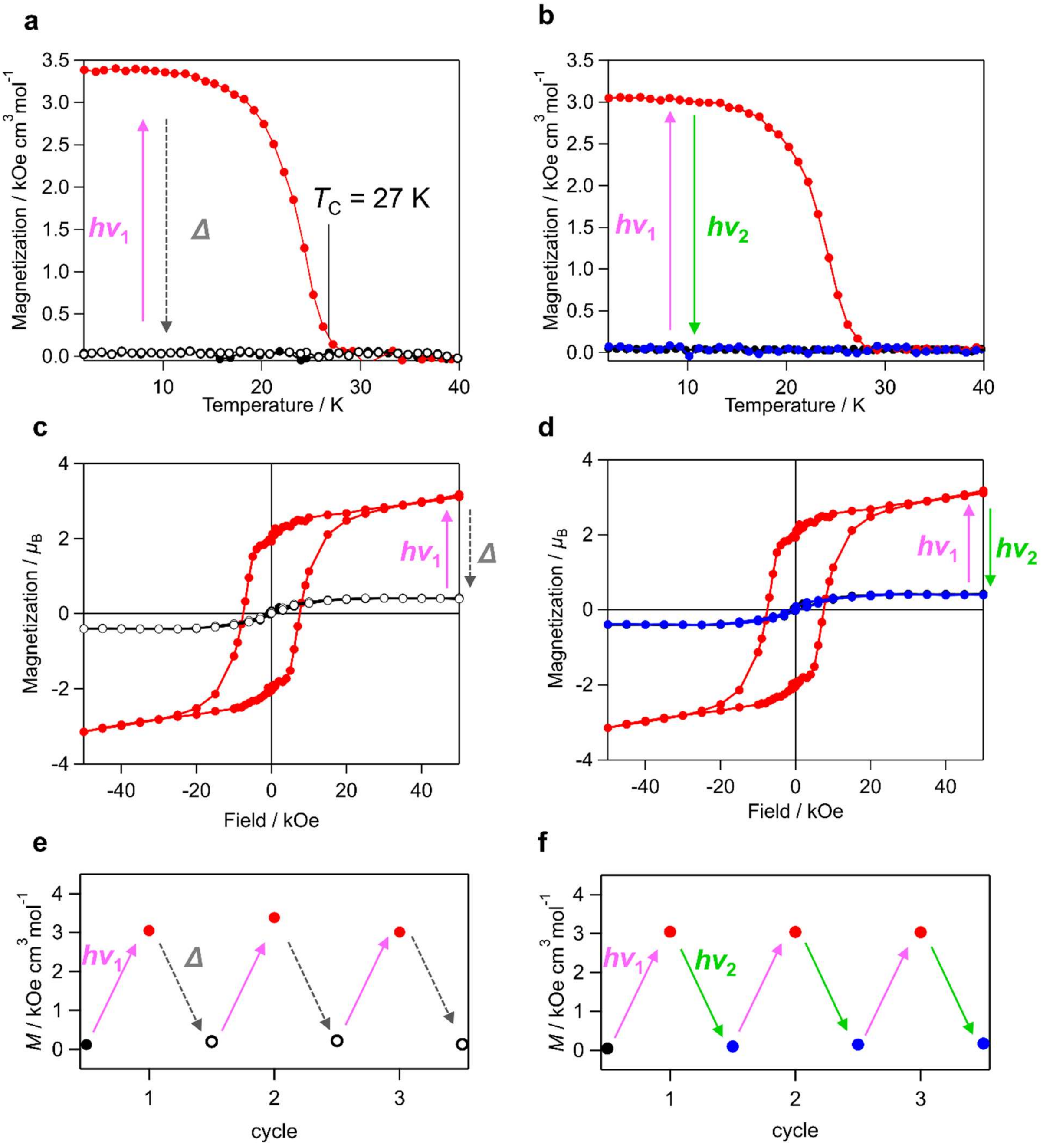

Supplementary Fig. 5. Photomagnetic measurements for CsCoW. Photo-thermal phase transition (left) and reverse-PIPT (right). **a, b** FCM curves under 20 Oe. **c, d** *M-H* curves at 2 K. **e, f** Magnetization vs cycle plots. Paramagnetic LT state (Black circle), photomagnetic PI state induced at 785 nm (240 mW cm^{-2}) and 3 K (red), which thermally relaxes (Δ) to the LT state above 100 K (open black circle). Photoexcitation at 532 nm (145 mW cm^{-2}) and 3 K of the photomagnetic PI state switches back the system to the paramagnetic LT state (blue).

Supplementary Note 5: Sub-ps time delay ΔOD map of CsCoW at 230 K and 100 K

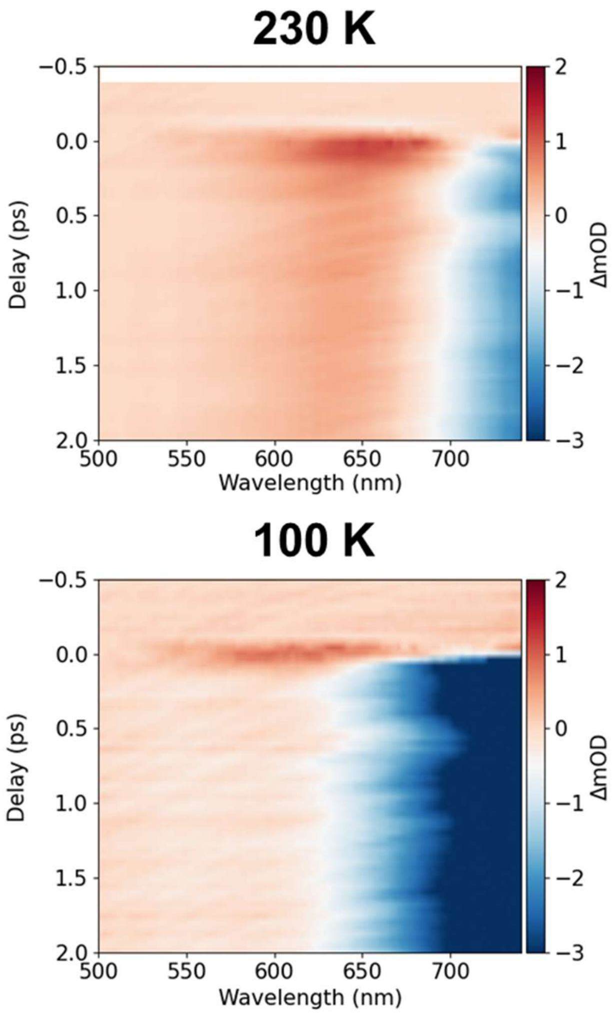

Supplementary Fig. 6. Time delay ΔmOD (ΔOD ×10^{-3}) maps. 230 K (top) and 100 K (bottom).

Supplementary Note 6: Data analysis of sub-ps time measurements

Optical data in the sub-ps timescale was fitted by separating two components which are the population and oscillation components.

(1) Population component

We fitted the evolution of the population Delta $\Delta OD_{Population}(t)$ by using the convolution of $\Delta OD_P(t)$ with the IRF function (S_{IRF}) :

$$\Delta OD_{Population}(t) = s_{IRF} \otimes \Delta OD_p(t) \quad \text{(S1)}$$

$$s_{IRF}(t) = \frac{1}{\sigma\sqrt{2\pi}} e^{-\frac{1}{2}\left(\frac{t-t_0}{\sigma}\right)^2} \quad \text{(S2)}$$

σ and t0 corresponds to the laser pulse width (~ 65 fs) and relative time delay corresponding to the time zero event. $\Delta OD_P(t)$ is composed of the two components which are $\Delta OD_{Decay}(t)$ and $\Delta OD_{Pop}(t)$:

$$\begin{aligned} \Delta OD_P(t) &= \Delta OD_{Decay}(t) + \Delta OD_{Pop}(t) \\ &= A_d.\exp\left(-\frac{t-t_0}{\tau_{Decay}}\right) + A_p.\left[1-\exp\left(-\frac{t-t_0}{\tau_{Decay}}\right)\right] + A_T\mathrm{t} + A_{CST} \quad \text{(S3)} \end{aligned}$$

A_d, A_p, τ_{Decay}, indicate the amplitude of decay function and population function, and the decay timescale. The linear function A_tt + A_{CST} represents the approximation of the population function by the thermo-elastic conversion.

Supplementary Table 1. Summary of the fit results at 600 nm and 700 nm.

	CoW	CsCoW			Average
Temperature	RT	RT	230 K	100 K	
Decay (600 nm) / fs	121 (4)	136 (5)	123 (3)	135 (5)	129 (8)
Decay (700 nm) / fs	115 (6)	137 (3)	139 (9)	115 (9)	127 (12)

(2) Oscillation component

In order to isolate the oscillations, we subtracted the fitted population components to the chirp corrected data and focus our attention to the residual signal after 300 fs (Supplementary Fig. 7). Because the time-zero artefact from the substrate does not impact the sample's signal as it does not display any photoinduced response 300 fs after photoexcitation. Supplementary Fig. 7a, 7b, 7c, and 7d corresponds to the transient 2D maps of the CoW, CsCoW at RT, 230 K and 100 K, respectively, after application of the data analysis previously explained. Supplementary Figure 8 corresponds to the computed global FFT of Supplementary Figs. 7, 9) is a zoom of Supplementary Fig. 8 between 500 to 620 nm. The frequency range was limited to 500 cm^{-1} due to the laser pulse width (65 fs ≈ 513 cm^{-1}). The FFT amplitudes around 700 nm in Supplementary Fig. 8 are much stronger than the one around 600 nm because the population signal is stronger. Overall, the signal for CsCoW at 230 K and 100 K are less stable compared with the 300 K ones because the cryogun might disturb the signal and the photoinduced signal is smaller (population and amplitude of the oscillations). From those results, around 600 and 700 nm, one can distinguish two predominant frequencies around 130 and 58cm^{-1}, that we assigned in Supplementary Table 2.

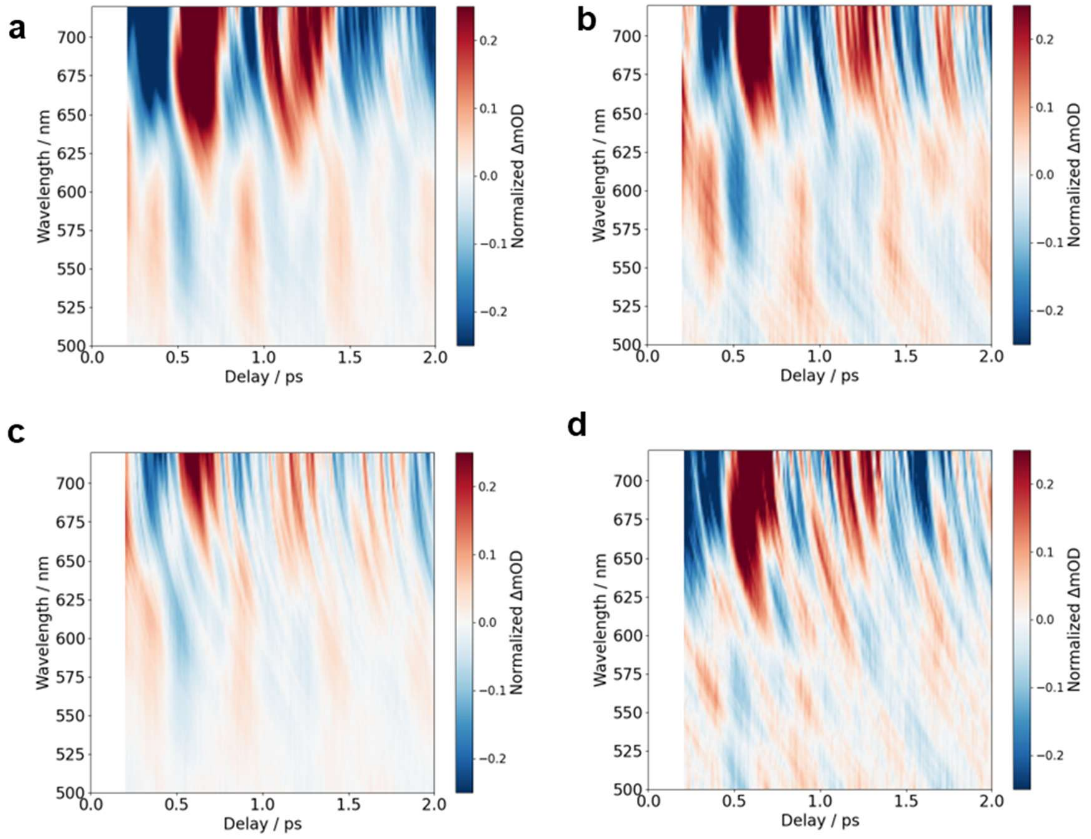

Supplementary Fig. 7. Oscillation components of the time-resolved 2D maps after removing the population fitting. **a** CoW at RT. **b, c, d** CsCoW at RT, 230 K, and 100 K, respectively.

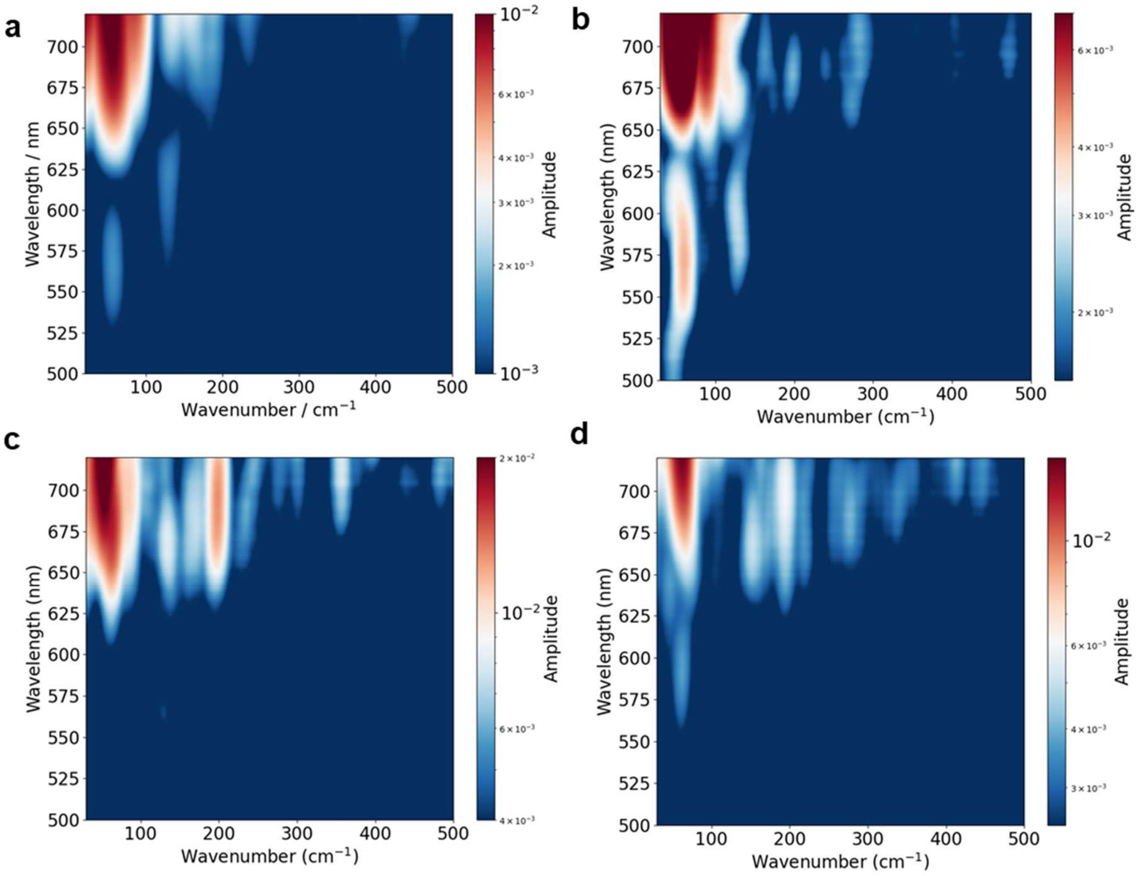

Supplementary Fig. 8. Global FFT maps for oscillation components. a CoW at RT. **b, c, d** CsCoW at RT, 230 K and 100 K, respectively, in the range of 500 nm - 720 nm.

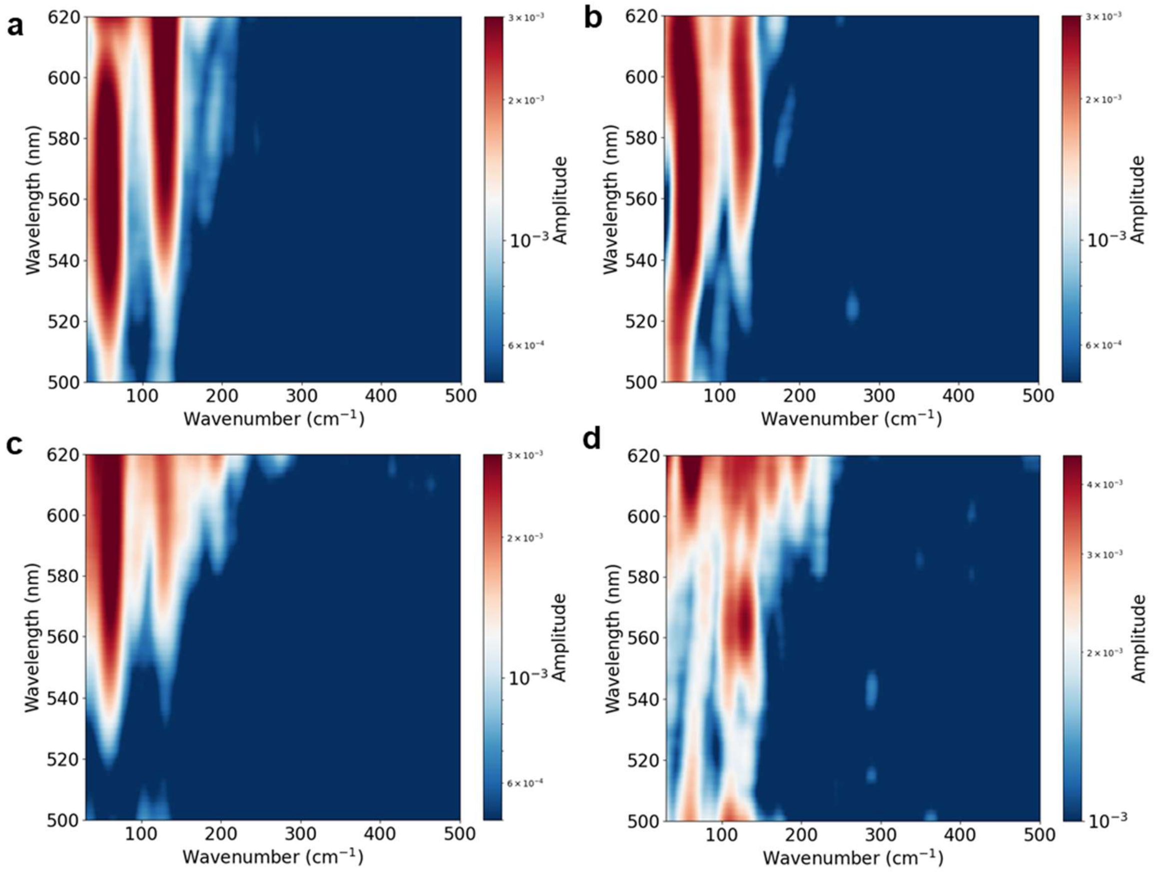

Supplementary Fig. 9. Global FFT maps for oscillation components. a CoW at RT. **b, c, d** CsCoW at RT, 230 K and 100 K, respectively, in the range of 500 nm - 620 nm.

Supplementary Table 2. Assigned frequencies of the main oscillations from the FFT maps at 600 nm and 700 nm.

	**CoW**	**CsCoW**			Average
Temperature	RT	RT	230 K	100 K	
Short Osc. / cm^{-1}	129 (3)	130 (4)	128 (2)	130 (4)	129 (2)
Long Osc. / cm^{-1}	55 (3)	60 (3)	62 (5)	53 (6)	58 (5)

The fit of the transient oscillating components were performed by using two damped oscillations with the following equation:

$$\Delta OD_{OSCfit} = \sum_{i=1}^{2} \Delta OD_{OSC}^{I} = \sum_{i=1}^{2} A_{osci}.\exp\left(-\frac{t}{\tau_{dampi}}\right).\cos(\omega_i t + \phi_i) \quad \text{(S4)}$$

Where A_{OSC}, τ_{damp}, ω, Φ indicate the amplitude, decay constant, oscillation period and phase of the damped oscillation. For the fit, we fixed the frequency of the oscillations based on the 2D FFT results (Supplementary Table 2). Since only the data of CsCoW at 100 K has stronger noise than other data which affect to oscillation components, we decided not to treat it for oscillation fitting.

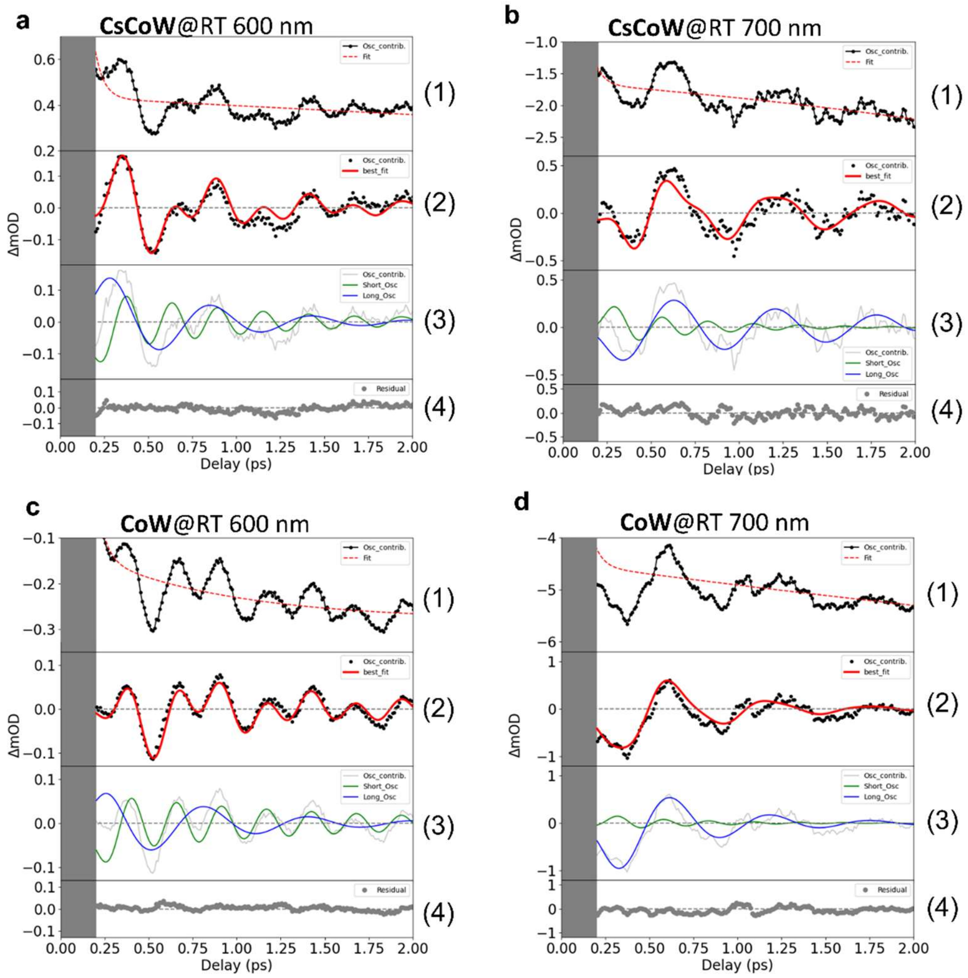

Supplementary Fig. 10. Oscillation fitting. a, b CsCoW at RT at 600 nm and 700 nm. **c, d** CoW at RT at 600 nm, and 700 nm. For each Supplementary Fig. (1) Transmission transient (**T**) in black dots, fit of the population in red dot line (**P**). (2) Transient oscillating part (**R_O**) in black dots (obtained with **R_O** = **T** — **P**), sum of the two oscillations in red (**F_O**). (3) The two damped oscillations (shoter: green, longer: blue) of **F_O**, compared with **R_O** in grey line. (4) Residual signal from **R_O** — **F_O**. The grey area corresponds to the time-zero artefact not taken into account.

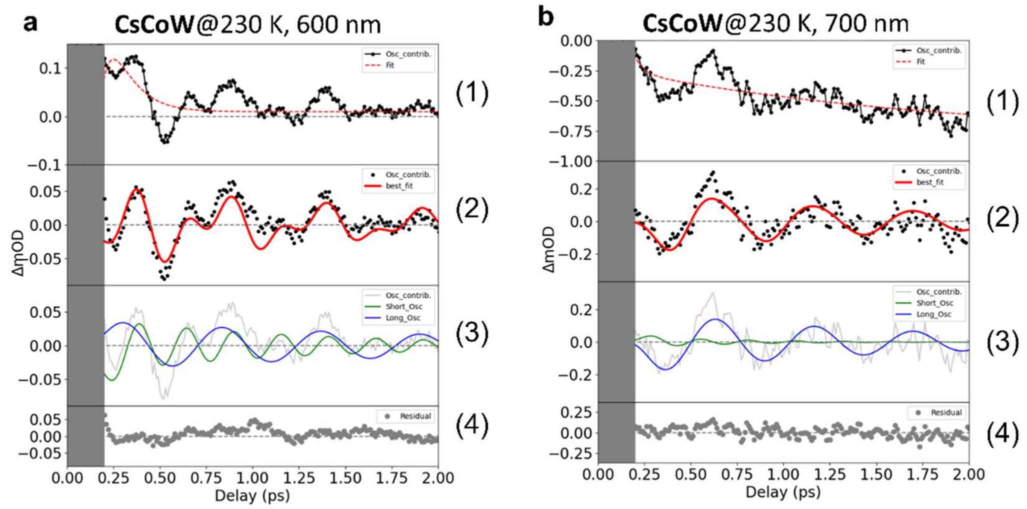

Supplementary Fig. 11. Oscillation fitting. a, b CsCoW at 230 K at 600 nm, and 700 nm. For each Supplementary Figs., (1) Transmission transient (T) in black dots, fit of the population in red dot line (P). (2) Transient oscillating part ($\mathbf{R_O}$) in black dots (obtained with $\mathbf{R_O} = \mathbf{R} - \mathbf{P}$), sum of the two oscillations in red ($\mathbf{F_O}$). (3) The two damped oscillations (shoter: green, longer: blue) of $\mathbf{F_O}$, compared with $\mathbf{R_O}$ in grey line. (4) Residual signal from $\mathbf{R_O} — \mathbf{F_O}$. The grey area corresponds to the time-zero artefact not taken into account.

Supplementary Note 7: DFT calculation

Phonon modes of 4-bromopyridine were observed in the 600–1600 cm^{-1} and around 3200 cm^{-1}.

Supplementary Table 3. Phonon modes obtained by DFT calculations in each electronic state of Co.

Co^{II}_{HS}* indicates the result of phonon mode calculation by using the structural optimization of Co^{II}_{LS}.

	Frequency (cm^{-1})					Frequency (cm^{-1})			
No.	Co^{III}_{LS}	Co^{II}_{LS}	Co^{II}_{HS}*	Co^{II}_{HS}	No.	Co^{III}_{LS}	Co^{II}_{LS}	Co^{II}_{HS}*	Co^{II}_{HS}
1	23.7	16.7	-70.9	14.7	22	302.7	216.1	224.4	226.5
2	23.7	17.4	-39.2	15.0	23	332.8	221.6	225.4	232.5
3	50.9	21.4	19.7	18.1	24	332.8	247.3	245.2	232.6
4	67.9	35.8	36.4	24.6	25	346.5	269.8	249.4	249.3
5	75.5	52.5	49.2	49.3	26	351.5	289.3	251.0	249.4
6	75.5	65.9	65.3	49.6	27	351.5	292.1	265.1	257.7
7	109.1	73.3	66.5	61.5	28	352.1	298.7	277.1	259.7
8	113.6	82.3	85.7	71.2	29	356.0	311.9	281.5	259.8
9	113.6	83.1	90.9	81.2	30	357.8	326.7	305.3	273.1
10	113.8	91.4	99.6	81.3	31	371.7	338.9	313.3	273.5
11	115.8	93.6	99.9	86.8	32	375.3	342.8	330.9	277.6
12	124.1	101.0	107.2	91.3	33	394.6	367.0	364.7	282.5
13	130.8	102.2	111.6	91.3	34	398.1	383.0	371.9	317.7
14	130.8	105.5	112.4	95.2	35	442.1	387.0	382.3	318.1
15	177.2	119.6	118.7	130.4	36	476.2	399.3	383.8	375.0
16	177.2	135.2	134.9	130.6	37	476.2	420.4	451.3	376.6
17	204.4	153.7	138.5	146.7	38	526.3	511.1	501.9	493.1
18	241.1	176.0	145.3	184.3	39	526.3	514.3	502.2	493.3
19	263.5	183.8	192.5	187.5	40	643.9	640.7	639.5	641.0
20	263.5	191.8	198.8	200.4	41	643.9	641.6	640.0	641.1
21	302.7	192.3	210.1	200.6	42	688.4	670.2	669.5	657.9

	Frequency (cm^{-1})					Frequency (cm^{-1})			
No.	Co^{III}_{LS}	Co^{II}_{LS}	$Co^{II}_{HS}{}^{*}$	Co^{II}_{HS}	No.	Co^{III}_{LS}	Co^{II}_{LS}	$Co^{II}_{HS}{}^{*}$	Co^{II}_{HS}
43	688.5	671.3	670.6	658.7	66	1301.4	1294.6	1286.9	1286.0
44	722.4	709.7	715.4	717.6	67	1301.4	1295.5	1288.0	1286.1
45	722.4	712.3	716.9	717.6	68	1398.8	1391.2	1380.7	1380.6
46	808.6	798.2	797.0	791.4	69	1398.8	1392.4	1381.0	1380.8
47	808.6	798.8	797.2	791.5	70	1460.5	1451.7	1436.0	1440.4
48	822.3	811.1	817.2	814.2	71	1462.4	1452.1	1437.2	1440.8
49	822.8	811.8	817.7	814.5	72	1549.6	1550.9	1525.7	1529.5
50	947.5	943.2	951.6	947.5	73	1549.6	1552.5	1526.0	1529.8
51	947.5	943.8	952.3	947.5	74	1579.6	1556.0	1526.3	1544.5
52	964.6	963.0	970.3	968.4	75	1580.0	1556.2	1527.3	1544.5
53	965.6	965.8	972.4	969.5	76	2095.2	2061.6	2031.3	2071.7
54	1007.7	995.0	997.5	978.0	77	2095.2	2062.9	2032.1	2073.4
55	1008.3	995.1	999.1	979.1	78	2099.3	2090.3	2067.6	2073.5
56	1034.6	1025.6	1016.1	1027.6	79	2105.1	2092.8	2068.5	2077.0
57	1039.9	1025.7	1017.0	1028.0	80	3109.7	3096.1	3068.1	3075.9
58	1073.7	1065.1	1054.1	1059.6	81	3109.8	3096.2	3068.5	3075.9
59	1073.8	1065.2	1054.1	1059.7	82	3110.0	3096.6	3068.5	3076.9
60	1087.8	1072.0	1063.7	1060.1	83	3110.0	3096.6	3068.8	3076.9
61	1087.8	1073.8	1065.0	1060.2	84	3136.0	3105.0	3078.5	3089.9
62	1202.8	1198.0	1193.7	1195.6	85	3136.0	3105.4	3078.7	3089.9
63	1204.7	1198.6	1194.2	1196.4	86	3138.0	3107.3	3081.0	3092.0
64	1251.4	1249.2	1216.1	1236.6	87	3138.7	3107.8	3081.3	3092.1
65	1251.4	1249.8	1216.6	1236.7					

a

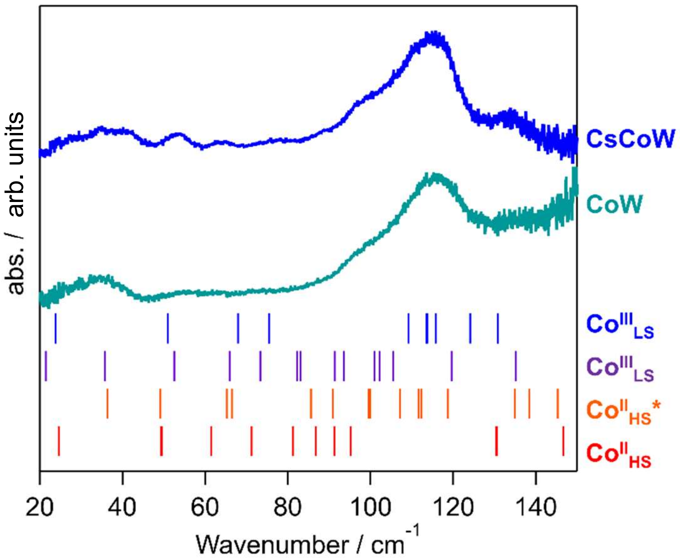

b

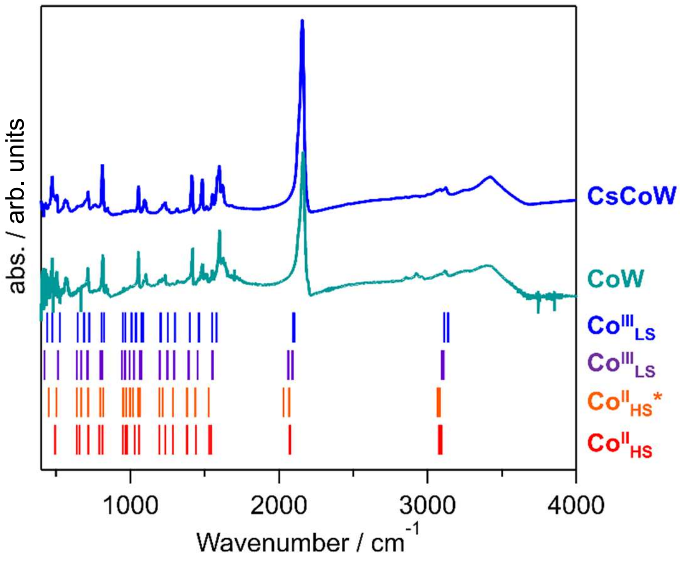

Supplementary Fig. 12. Vibrational spectra. **a** THz absorbance spectra at RT. **b** IR spectra at RT. Blue and light blue lines indicate the experimental data of the LT state of CsCoW and CoW, whereas blue, purple, orange, and red bars correspond to calculated phonon modes of Co^{III}_{LS}, Co^{II}_{LS}, $Co^{II}_{HS}{}^{*}$, and Co^{II}_{HS}, respectively. $Co^{II}_{HS}{}^{*}$ represents the calculated frequencies after the structural optimization as Co^{II}_{LS}.

Supplementary Fig. 13 shows the results of band energy calculations of the LT state, obtained by DFT. The highest occupied crystalline orbital (HOCO) has a strong W character, while the lowest unoccupied crystalline orbital (LUCO) has a strong Co character. Excitation in the NIR region corresponds to the W → Co optical transition, which is driving the CTIST process. Investigating the STICT process requires using a Co → Co centered transitions, which is found around 5 eV (250 nm). We could not explore the STICT mechanism, as our pump beam cannot reach this excitation wavelength.

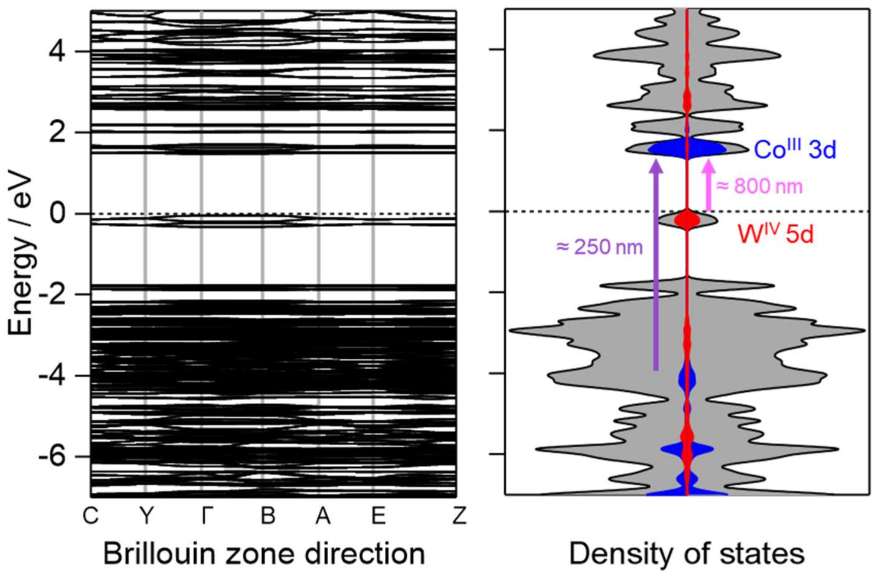

Supplementary Fig. 13. First-principles calculations of CoW. Band structure (left) and density of states (DOS) (right) of CoW from −7 eV to 5 eV reproduced from reference 38. Grey, blue, and red areas represent total DOS and partial DOS for Co^{III} 3d and W^{IV} 5d, respectively. The W → Co transition is around 1.5 eV (pink arrow ≃800 nm), while the Co → Co transition is around 5 eV (purple arrow ≃250 nm).

Supplementary Note 8: Data analysis of long-picosecond time scale

The fitting of CsCoW at RT and 230 K was conducted with two population functions. The first population describes the appearance of photoexcitated local PI state within 40 to 60 ps relying on the sample initial temperature. While the second population appears after the first one and corresponds to the lattice relaxation.

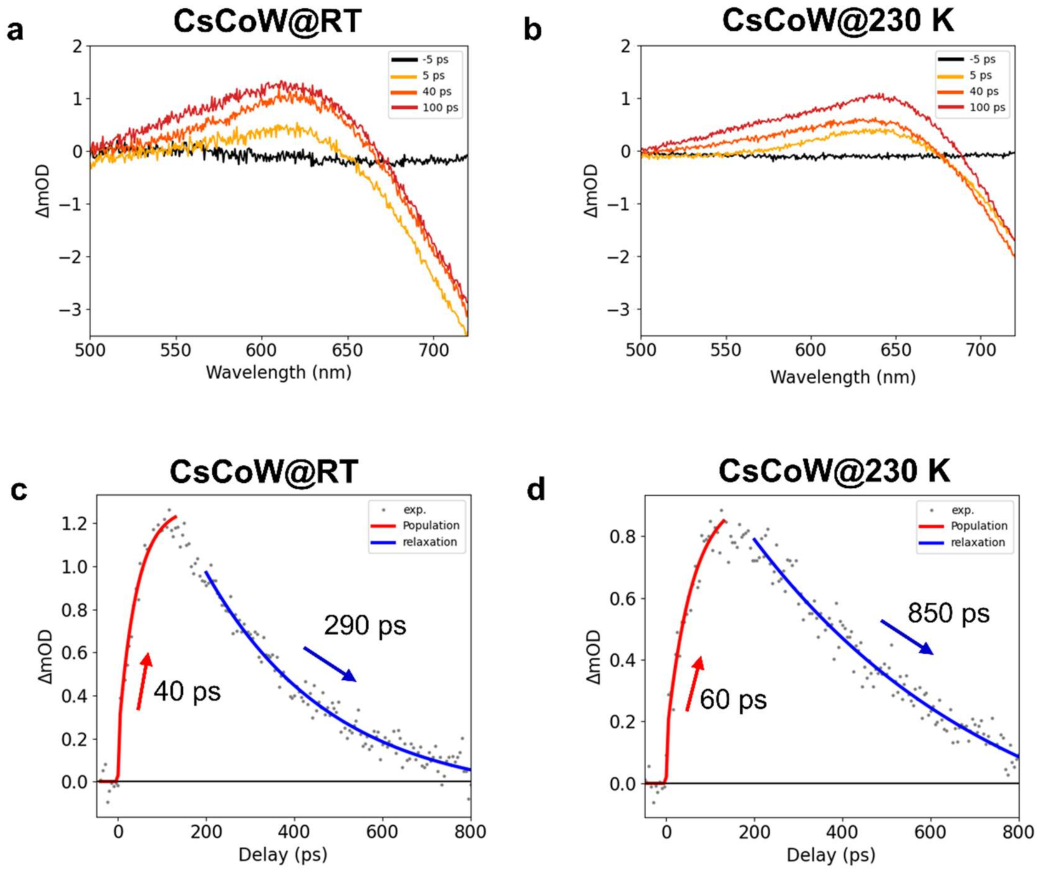

Supplementary Fig. 14. Time-resolved optical spectroscopy in ps timescale. a, b Photoinduced spectral change of CsCoW for four different delays at RT, and 230 K. **c, d** Transient dynamics at 580 nm (±20 nm) of CsCoW at RT c and 230 K, respectively. The measured dataset are in gray dot, whereas the red and blue lines indicate fitting of the population and relaxation, respectively.

Supplementary Note 9: Gaussian fitting for spectral trace

The Gaussian fitting for CsCoW and CoW at 1.5 ps and 100 ps was conducted with two Gaussian functions to confirm the existence of the PI state, which is similar to the HT state.

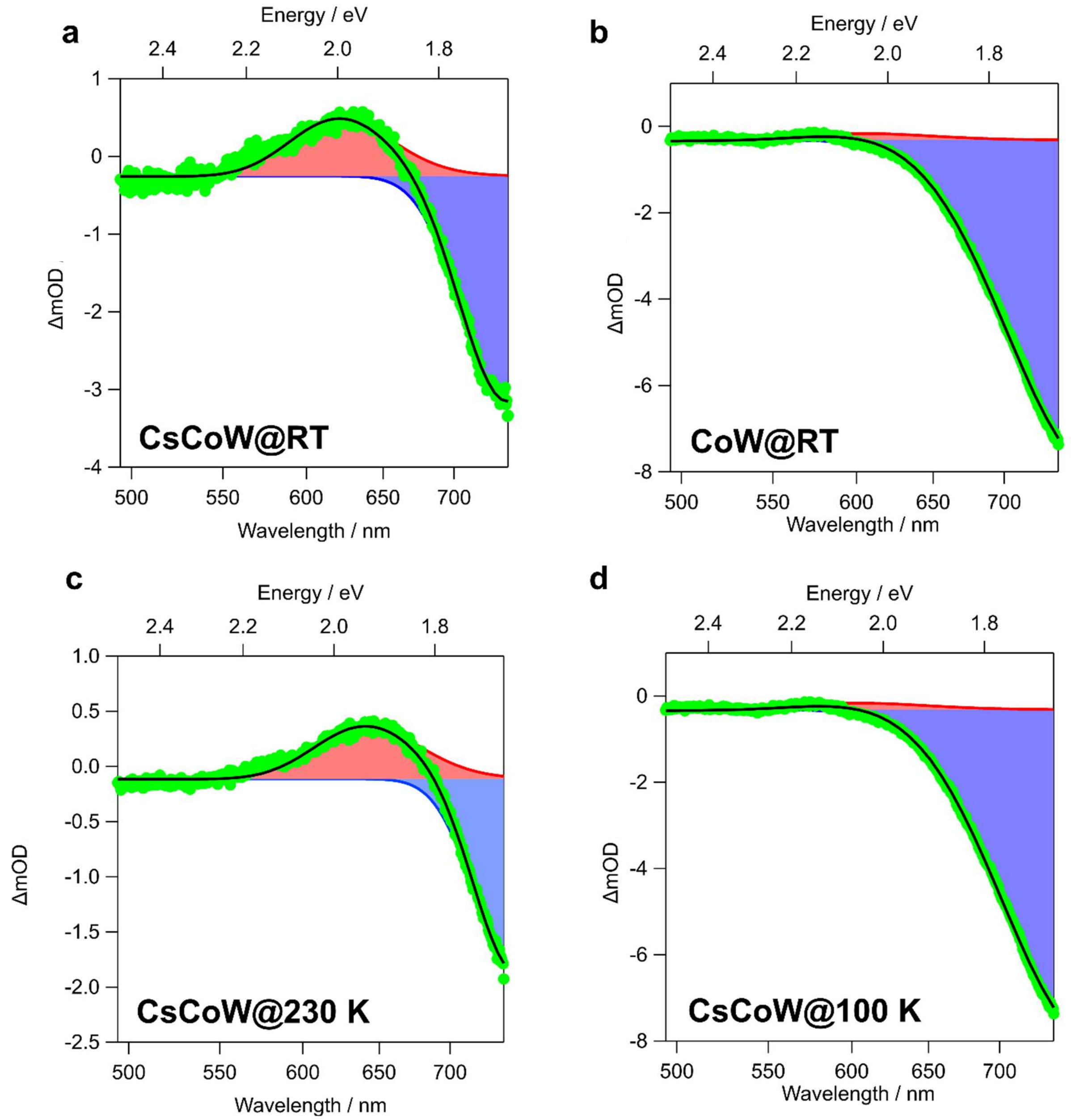

Supplementary Fig. 15. Spectral trace at 1.5 ps (± 0.1 ps) with Gaussian fitting. a CsCoW at RT. **b** CoW at RT. **c** CsCoW at 230 K. **d** CsCoW at 100 K. Green, black, red, and blue lines indicate the experimental data, fitting result, and Gaussian of the HT and LT state in the fit, respectively.

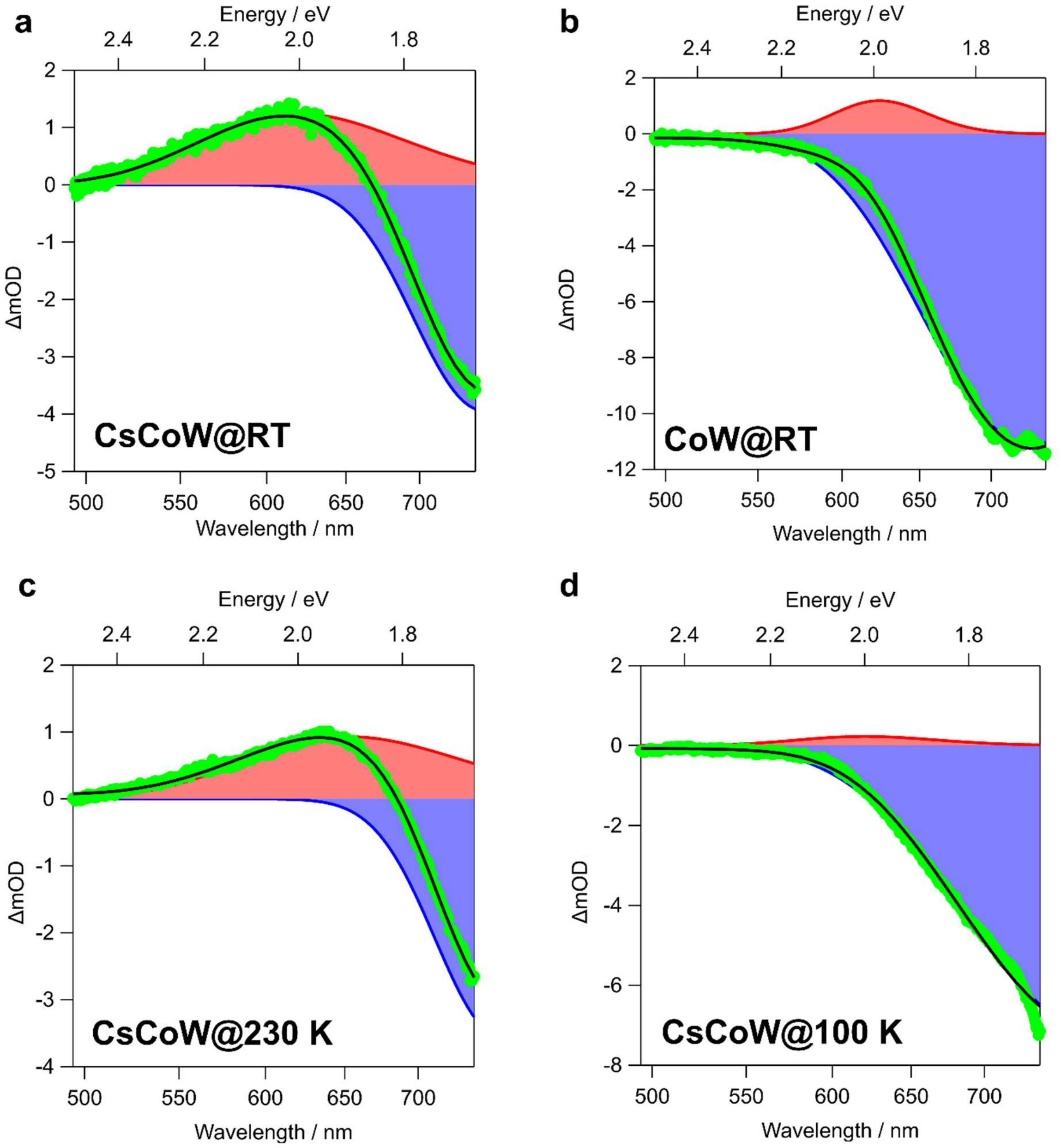

Supplementary Fig. S16. Spectral trace at 100 ps (± 10 ps) with Gaussian fitting. a CsCoW at RT. **b** CoW at RT. **c** CsCoW at 230 K. **d** CsCoW at 100 K. Green, black, red, and blue lines indicate the experimental data, fitting result, and Gaussian of the HT and LT state in the fit, respectively.

Supplementary Table 4. Fitting parameters of two Gaussian functions in Supplementary Fig. 14.

1.5 ps	sample	CsCoW			CoW
	Temperature	RT	230 K	100 K	RT
LT state	Position (eV)	1.67	1.65	1.60	1.60
	Height	-2.90	-1.78	-4.72	-7.69
	FWHM	0.19	0.17	0.36	0.37
PI state (HT state)	Position (eV)	2.00	1.93	1.85	2.05
	Height	0.74	0.48	0.10	0.15
	FWHM	0.25	0.25	0.20	0.33

Supplementary Table 5. Fitting parameters of two Gaussian functions in Supplementary Fig. 15.

100 ps	sample	CsCoW			CoW
	Temperature	RT	230 K	100 K	RT
LT state	Position (eV)	1.66	1.63	1.57	1.70
	Height	-3.94	-3.56	-7.18	-11.1
	FWHM	0.28	0.26	0.54	0.46
PI state (HT state)	Position (eV)	2.00	1.90	2.00	1.99
	Height	1.25	0.93	0.23	1.19
	FWHM	0.49	0.50	0.33	0.23

Supplementary Note 10: Fluence dependence at RT

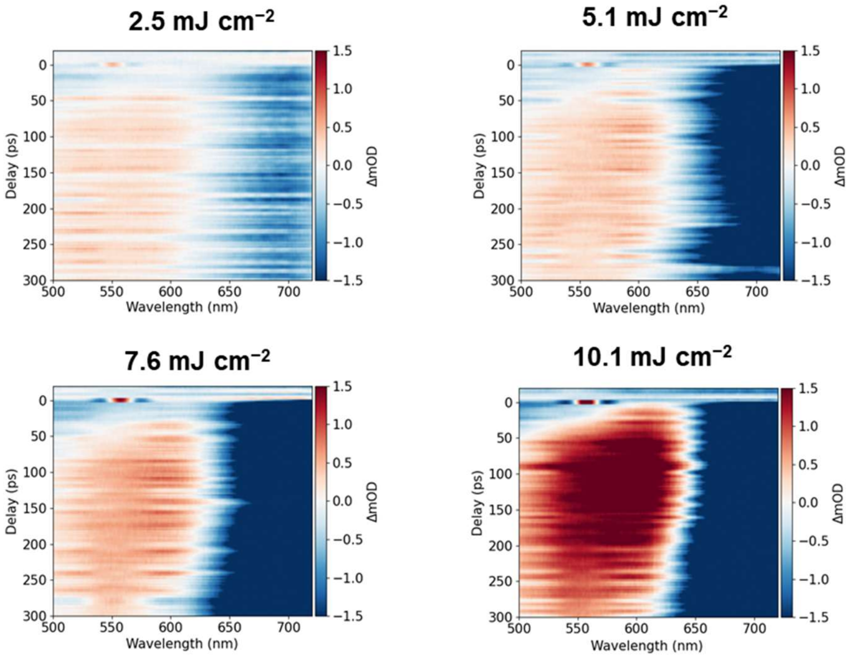

Supplementary Fig. 17. Delay vs wavelength vs ΔmOD maps with different fluences for CsCoW at RT.

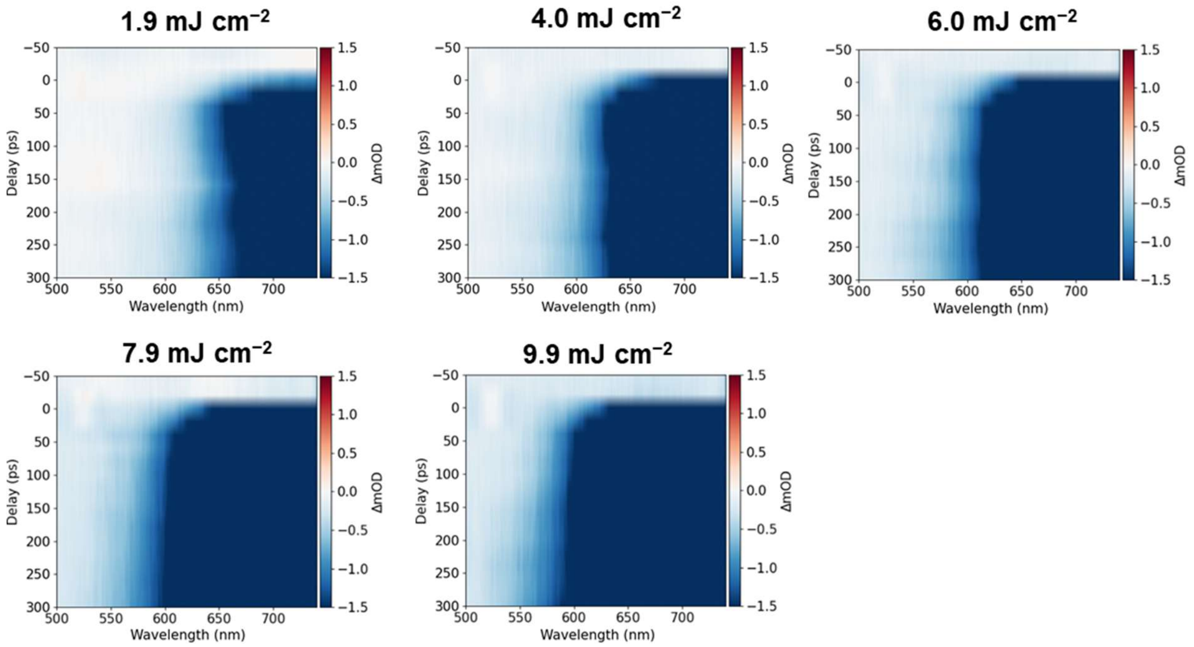

Supplementary Fig. 18. Delay vs wavelength vs ΔmOD maps with different fluences for CoW at RT.
