# Supplementary material for: Ultrafast charge-transfer-induced spin transition in cobalt-tungstate molecular photomagnets
Source: Nat Commun. 2025 Jun 6;16:5012. doi: 10.1038/s41467-025-60401-4 (PMC12144094; doi:10.1038/s41467-025-60401-4)
Supplement: Supplementary file 2 — Description of Additional Supplementary Files [file 41467_2025_60401_MOESM2_ESM.pdf]

Description of Additional Supplementary Files:

Supplementary Movie 1: Movie of the phonon modes of the breathing mode involving Co–N bond elongation (right) and the torsion modes representing distortions of Co–NC–W bridges (left).
