# Supplementary figures and images for: Ultrafast charge-transfer-induced spin transition in cobalt-tungstate molecular photomagnets
Source: Nat Commun. 2025 Jun 6;16:5012. doi: 10.1038/s41467-025-60401-4 (PMC12144094; doi:10.1038/s41467-025-60401-4)

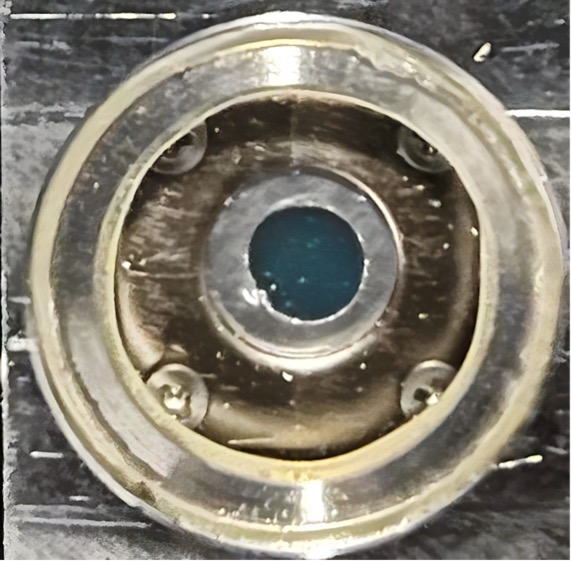

Supplement: Supplementary file 5 — Source Data [file 41467_2025_60401_MOESM5_ESM.zip › Source_Data_files/Photoinduced_UV_vis_NIR_spectra_CsCoW/LTstate.JPG]

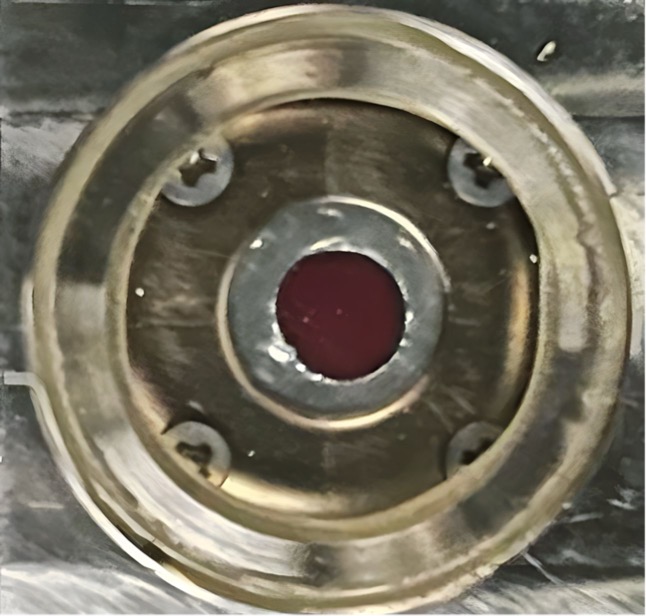

Supplement: Supplementary file 5 — Source Data [file 41467_2025_60401_MOESM5_ESM.zip › Source_Data_files/Photoinduced_UV_vis_NIR_spectra_CsCoW/PIstate.JPG]
